# LncRNA CASC19 promotes pancreatic cancer progression by increasing PSPC1 protein stability and facilitating the oncogenic PSPC1/ β-Catenin pathway

**DOI:** 10.1186/s10020-025-01363-7

**Published:** 2025-09-29

**Authors:** Moumita Mukherjee, Swati Ghosh, Atanu Maity, Sukanta Ray, Hemabha Saha, Ranjit Prasad Bahadur, Srikanta Goswami

**Affiliations:** 1 Biotechnology Research and Innovation Council, National Institute of Biomedical Genomics (BRIC-NIBMG), 741251 Kalyani, India; 2 Regional Centre for Biotechnology, 3rd Milestone, Faridabad-Gurugram Expressway, Faridabad, 121001 India; 3 Bioinformatics Centre, Department of Bioscience and Biotechnology, Indian Institute of Technology Kharagpur, Kharagpur, 721302 India; 4 Institute of Post Graduate Medical Education & Research, West Bengal 700020 Kolkata, India; 5 Computational Structural Biology Laboratory, Department of Bioscience and Biotechnology, Indian Institute of Technology Kharagpur, Kharagpur, 721302 India

**Keywords:** Pancreatic cancer, LncRNA, CASC19, PSPC1, Βeta-catenin, Metastasis

## Abstract

**Background:**

Pancreatic cancer, a highly lethal malignancy, is influenced by complex lncRNA-mediated gene expression. This study identified CASC19 as a significantly overexpressed lncRNA with high oncogenic potential in pancreatic cancer, aiming to uncover its molecular mechanism in tumor progression.

**Methods:**

CASC19 expression was evaluated by qRT-PCR. Proliferation abilities of CASC19 were evaluated by MTT assay, cell cycle and apoptosis assay, upon CASC19 overexpression and knockdown. Whereas its metastatic potential was evaluated by wound healing, migration and invasion assay. The effect of CASC19 on cellular transcriptome was also examined by RNA sequencing after CASC19 overexpression and silencing. Subcellular localization of the lncRNA was examined by subcellular fractionation followed by qRT-PCR. To find out the molecular mechanism of lncRNA function, RNA-pull down, mass-spectrometry, immunoprecipitation, protein stability and ubiquitination assays were done.

**Results:**

High CASC19 expression was found in pancreatic tumor tissues and multiple pancreatic cancer cell lines. In-vitro loss and gain-of-function experiments showed that CASC19 is an oncogenic lncRNA promoting proliferation and metastasis of pancreatic cancer, while inhibiting apoptosis. CASC19 was also found to regulate global transcriptome of pancreatic cancer cells, affecting pathways like TGF-β signaling pathway, β-Catenin-TCF complex assembly, etc. CASC19 was localized to nucleus of pancreatic cancer cells and was identified to interact with PSPC1, a metastatic reprogramming protein. The interaction results in prevention of ubiquitin-mediated degradation of PSPC1. Increased availability of PSPC1, in turn, potentiates nuclear retention of β-Catenin which ultimately triggers pancreatic cancer progression.

**Conclusion:**

Our findings elaborate the mechanism of CASC19 mediated tumorigenesis in pancreatic cancer, highlighting the role of PSPC1 in the process. Targeting CASC19/PSPC1/β-Catenin axis could be a novel approach to impede the progression of the disease.

**Supplementary Information:**

The online version contains supplementary material available at 10.1186/s10020-025-01363-7.

##  Introduction

Pancreatic cancer is one of the most aggressive cancer types worldwide and it has been estimated to be the next leading cause of death due to malignancy after lung cancer by 2030 in the United States (Gordon-Dseagu et al. 2018). As it is diagnosed at very advanced stage, despite all the advances in treatment, patients exhibit a 5-year survival rate of only 10% (Siegel et al. 2021). Patients with resectable tumours generally have more prolonged survival than patients with unresectable tumours, but only 10–20% of the patients are diagnosed at a stage amenable to resection for lack of atypical symptoms and early diagnostic biomarkers (Poruk et al. 2013; Yang et al. 2020). Even after curative resection, cancer recurs in majority of the patients and about 85–90% of the cases are diagnosed with micro-metastasis or distant metastasis (Das and Batra 2015). Hence, exploring the altered milieu in pancreatic cancer and the underlying molecular and cellular mechanisms that contribute to the aggressiveness of the disease is essential for identifying potential therapeutic targets for the disease.

Massive explosion of global transcriptomic data has remodeled the mRNA-centric paradigm of transcriptome by the omnipresent noncoding RNAs, of which long noncoding RNAs (lncRNAs) are most noteworthy in terms of their tissue-specific expression pattern and redundant functionality. LncRNAs are > 200nt non-coding transcripts transcribed by RNA Polymerase-II and individual lncRNAs manifest distinct RNA elements as RNA sequences or structures, critical for their functioning (Jarroux et al. 2017; Kopp and Mendell 2018). Flexibility of these non-coding transcripts and their ability to fold into complex higher order structures enable them to form interactions with DNA, RNA and protein molecules, portraying their massive potential to modulate complex regulatory circuits of cellular machinery (Guttman and Rinn 2012; Schmitz et al. 2016).

Emerging evidence suggests a diverse mode of action for lncRNAs. One of their familiar functions is regulation of transcription through epigenetic amendment like histone modification and chromatin remodeling (Lee 2012). LncRNAs also deal with stability and localization of several crucial proteins to oversee the entire central dogma process (Das and Batra 2015). LncRNAs transcribed from enhancer regions can have a repressive effect on the gene transcribed from the same locus by promoting enhancer looping. Additionally, a group of lncRNAs have a crucial role in sponging microRNAs and titrating the inhibitory effect of microRNAs on their target genes (competing endogenous RNA (ceRNA) hypothesis) (Tay et al. 2014). Altered lncRNA profile can have an impact on entire tumorigenesis process along with its hallmarks like metastasis, angiogenesis, proliferation, apoptosis and tumor suppression (Bhan et al. 2017). Multiple studies have assigned intricate functions to annotated lncRNAs. However, functions of unannotated and less explored annotated lncRNAs are still an important question to ask. Following that, the molecular characterization and functional implication of some lncRNAs which are already reported to be deregulated in pancreatic cancer, also demands deep exploration.

While exploring the deregulated lncRNA profile in pancreatic cancer, we found a cardinal lncRNA, Cancer Susceptibility Candidate 19 (CASC19) which has been shown to be deregulated in several other cancer types such as gastric cancer, colorectal cancer, non-small cell lung carcinoma, cervical cancer and nasopharyngeal carcinoma (Liu et al. 2020, 2021; Qu et al. 2019; Wang et al. 2019a, b, 2020, 2023a, b, c; Zhao et al. 2019). CASC19 is a 324-nucleotide long lncRNA (NR_120364) encoded in a chromosomal position 8q24.21 which also encodes the oncogenic protein MYC and an oncogenic lncRNA PVT1 (Wilson and Kanhere 2021; Zhang et al. 2016). In this study, we aim to inquire about the oncogenic role of CASC19 in pancreatic cancer proliferation, migration, cell cycle and apoptosis. Notably, we revealed the potential interaction between CASC19 and a metastatic reprogramming protein PSPC1 (paraspeckle component 1), which is known to be involved in the nucleocytoplasmic shuttling of oncoproteins like β-catenin to promote tumor progression (Lang et al. 2019; Nazemalhosseini Mojarad et al. 2015). Hereby, we find that CASC19 enhances the PSPC1 protein stability and thus potentiates the PSPC1-mediated nuclear translocation of β-catenin to promote pancreatic cancer progression. Thus, the positive correlation of CASC19 and PSPC1 expression and the CASC19/PSPC1/β-catenin axis seems to be a promising therapeutic target in pancreatic cancer treatment.

##  Methods

### Patient samples

Pancreatic tumor tissues (*n* = 17) and adjacent normal pancreatic tissues (*n* = 17) were collected from Institute of Post Graduate Medical Education & Research, Kolkata. All the tissues were undergone pathological confirmation of malignancy and frozen in RNA later at − 80 °C until further use. All the samples were collected upon ethical approval from the concerned hospital and after receiving informed consent from the patients or their families.

### Cell lines and cell culture conditions

Human pancreatic cancer cell lines AsPc1, MiaPaCa2 and BxPc-3 were kindly provided by Dr. S. Senapati from BRIC-Institute of Life Sciences (Odisha, India). Panc-1 pancreatic cancer cell line was obtained from cell repository of BRIC-National Centre for Cell Science (NCCS) (Maharashtra, India). Human pancreatic normal ductal epithelial cell line hTERT-HPNE and another pancreatic cancer cell line CAPAN-2 were purchased from the American Type Culture Collection (ATCC) (Manassas, VA, USA). HPNE cell Line was grown in 75% DMEM with 25% Medium M3 Base (INCELL Corporation LLC, San Antonio, TX, USA), 5% FBS, 10 ng/ml human recombinant EGF, 5.5 mM d-glucose (1 g/L), and 750 ng/ml puromycin. BxPc-3 cell line was cultured in RPMI1640 medium (Gibco; Thermo Fisher Scientific, USA) with 10% FBS and 1% antibiotic-antimycotic solution, whereas the other pancreatic cancer cell lines were maintained in Dulbecco’s modified Eagle’s medium (DMEM) (Gibco; Thermo Fisher Scientific, USA) supplemented with 10% fetal bovine serum (FBS) and 1% antibiotic-antimycotic solution. All the cells were maintained in a 5% CO2 saturated humidified incubator at 37 °C and the cells were routinely monitored to verify the absence of mycoplasma contamination.

### Transient and stable transfections

For CASC19 overexpression, the human full length CASC19 was cloned into mammalian expression vector pcDNA3.1(+). The vector pcDNA3.1 with the inserted sequence along with the empty vector were transiently transfected into pancreatic cancer cell MIAPaCa-2 using the transfection reagent Lipofectamine 3000 (Invitrogen, Carlsbad, CA, USA) according to manufacturer’s instruction. To generate CASC19 overexpressing stable cell line, transiently transfected MIAPaCa-2 cells were selected using 1.5 µg/mL of geneticin for 14 days. For knockdown, CASC19 DsiRNAs and a scramble DsiRNA (IDT, catalog no.: 51-01-14-03) were purchased from IDT (Integrated DNA Technologies, USA). PSPC1 siRNAs (used in 1:1 ratio) and its negative control siRNA (Qiagen, Cat No.: SI03650318) were purchased from Qiagen (QIAGEN, Valencia, CA). All the siRNA sequences are given in Supplementary Table-S1. All the transient transfections were performed for 48 h, and post-transfection gene expression levels were assessed by qRT-PCR.

### RNA isolation and quantitative real-time PCR

Total RNA from the cell lines was extracted using QIAzol^®^ lysis reagent (QIAGEN, Valencia, CA) according to the manufacturer’s protocol. For total RNA extraction from patient tissues, 20 mg of tissue sections for each sample were processed using the Qiagen All-Prep DNA/RNA/miRNA isolation kit following the manufacturer’s instructions. RNA quantification was done by ND 8000 multi-channel spectrophotometer (Thermo Fisher Scientific). For cDNA synthesis, reverse transcription was done with QuantiNova Reverse Transcription Kit (QIAGEN, Valencia, CA) using 500ng of RNA under the reaction condition at 25 °C for 3 min, 45 °C for 20 min, 85 °C for 5 min and final holding step on ice. Real time PCR was done using PowerUp™ SYBR™ Green Master Mix (Applied Biosystems, Thermo Fisher Scientific, US). Relative gene expression was measured using 2 − ΔΔCq method using PMM1 as endogenous control. Primer sequences are provided in the Supplementary Table-S2.

### Immunoblotting

Total protein from desired cell lines were isolated using RIPA buffer supplemented with protease inhibitors. Pierce BCA protein assay kit was used for protein concentration estimation according to the manufacturer’s protocol. SDS-PAGE gel was run for electrophoresis and proteins were then transferred to PVDF membrane (Merck Millipore). The proteins in the membrane were then blocked with 5% non-fat dry milk in TBST for 1.5 h. Then the PVDF membrane was washed and incubated overnight with primary antibody at 40 C. Next day, the membrane was re-incubated with secondary antibody for 1 h. The protein bands were detected by Clarity Western ECL Substrate (BioRad) and Image Lab software was used for the densitometric analysis. Refer to Supplementary Table-S3 for a list of the antibodies used in this research.

### MTT cell proliferation assay

Post lncRNA overexpression and knockdown, cell proliferation was assessed by MTT (3-(4,5-dimethylthiazol-2-yl)−2,5-diphenyltetrazolium bromide) assay. Cells were seeded in 96-well plate at an initial density of 2 × 10^3^ cells/well and proliferation was measured at 0 h, 24 h, 48 h and 72 h time points. MTT reagent was added at a final concentration of 0.5 mg/ml and DMSO was added after 4 h of incubation to dissolve the formazan. The absorbance was then measured by a spectrophotometer at 570 nm wavelength.

### Cell cycle assay

Stable and transiently transfected cells were counted and fixed with 75% ethanol overnight at −20 °C. Then the cells were washed with PBS and stained with Ribonuclease A added propidium iodide (20 µg/ml) staining solution at room temperature in dark for 30 min before analysis. The cell cycle distribution was examined through flow cytometry (BD-FACS-Aria™-Fusion Flow Cytometer, USA).

### Apoptosis assay

For apoptosis assay, Annexin V-FITC Apoptosis kit (APOAF, sigma) was used following manufacturer’s instructions using BD-FACS-Aria™-Fusion Flow Cytometer (BD Biosciences, USA). With this flowcytometric dual staining method, the percentage of early and late apoptotic cells were determined in lncRNA overexpressing and knockdown cells.

### Wound healing assay

For evaluation of two-dimensional cell migration, wound-healing assay was used. Cells were seeded in 6-well plates and cultured in 0.5% FBS media until 80–90% confluency. Then, the cell monolayer was scratched with a 200uL microtip and observed healing of the scratched area under microscope at 0 h, 24 h and 48 h time points. At least three fields were photographed for each plate and analyzed with ImageJ software.

### Trans-well migration and invasion assay

For migration assay, transfected cells were harvested in 24-well trans-well plates with 8-µm pore inserts (Avantor Biosciences). Cell suspension containing 2 × 10^4^ cells in 200ul serum free media were seeded in the upper compartment and 800ul 10% FBS containing complete media was added to the lower chamber as a chemoattractant. After 24 h, migrated cells to the bottom side were fixed with 4% paraformaldehyde, whereas the remaining cells in the upper compartment were wiped off with a cotton swab. Fixed cells were then permeabilized with ice-cold methanol and stained with 0.2% crystal violet staining for 30 min. To assess the invasion property of the cells, Matrigel-coated inserts were used as invasion chamber (Corning) with a similar protocol to migration assay. Migratory and invasive cells were observed (200x magnification) under an inverted light microscope and cell numbers in the captured five random fields were quantified using ImageJ software.

### Subcellular fractionation

Nucleo-cytoplasmic fractionation of the MIAPaCa-2 cell lines was done using the NE-PER Nuclear and Cytoplasmic Extraction kit (Thermo Fisher Scientific) according to the manufacturer’s protocol. To evaluate the target RNA expression, RNA was extracted from both the cytosolic and nuclear part and subjected to qRT-PCR. GAPDH and β-actin were used as cytoplasmic control and U6, GAPDH intron, MALAT1 and Lamin were used as nuclear control.

### RNA sequencing and analysis

Total RNA was extracted using Trizol method from the lncRNA overexpressing and knockdown cell lines along with their respective control groups and total mRNA sequencing was done using Illumina NovaSeq 6000. Quality assessment was carried out using Agilent Bio-analyser 2100 with Agilent nano kit and library preparation was performed by TruSeq Stranded Total RNA Library prep kit. After the RNA sequencing, FastQ files were aligned to reference genome from NCBI using DRAGEN RNA aligner. The raw count matrix was generated from the quantification files using ‘txtimport’ tool. Log2 Fold change comparison between the groups was performed using ‘DESeq2’ and significantly expressed genes were selected with log2foldchange ≥ +/- 1.5 & *p*-value < 0.05.

### RNA pull down and mass-spectrometry (MS) assay

Sense and antisense transcripts of CASC19 were transcribed in vitro using the TranscriptAid T7 High Yield Transcription Kit (Thermo Scientific). For the in vitro transcription, sense and antisense CASC19 were cloned in pcDNA 3.1 under the T7 promoter. The constructs were then linearized with the restriction enzyme at the 3’ end of the insert, gel purified and used as template for the transcription reaction. Pierce™ RNA 3’ End Desthiobiotinylation Kit was used for biotin labelling of the sense and antisense transcripts of CASC19. Biotinylated RNA probes were then purified and used for RNA pull down with Pierce Magnetic RNA-Protein Pull-Down Kit according to the manufacturer’s protocol (in accordance with the manufacturer’s instructions) using nuclear lysate of MIAPaCa-2 cell. Finally, eluted proteins associated with the transcripts were identified by mass spectrometry (MS) and western blot.

### Molecular docking

The structure of the lncRNA CASC19 was modelled using trRosettaRNA webserver (Wang et al. 2023a, b, c) which performs homology modelling using templates from RNAcentral database (The et al. 2017) to build the model. Structure of PSPC1 was obtained from protein data bank (PDB ID: 5IFN) (Berman et al. 2000). From the crystal structure of human PSPC1 (homodimer), one 260 residue long monomer was extracted. The modeled structure of lncRNA and the protein are docked using two docking programs, HDOCK (Yan et al. 2017) and HADDOCK (Honorato et al. 2024) using their default set of parameters. The residues present in the dimer interface of PSPC1 were considered as binding residues for docking. For the lncRNA we have considered the complete structure as probable binding site. The best predicted structures form each program were selected based on docking score and were analyzed. After careful inspection of overall compactness of the structure, surface-area of the binding interface best docked structure was selected. 2D interaction diagram of the residue-level interaction between PSPC1 and lncRNA was generate using Ligplot (Wallace et al. 1995). PyMol (De Lano 2002) was used for structure visualization and image rendering.

### RNA immunoprecipitation (RIP) assay

Cells were harvested in 10 cm culture dishes. Approximately 1 × 10^7^cells were harvested and lysed in 0.5mL RIP lysis buffer supplemented with RNAse and protease inhibitors. MIAPaCa-2 cell lysates were incubated overnight with IgG, anti-PSPC1 and anti- β-catenin antibody at 4 degrees. The antibody-protein complexes were pulled with A + G dynabeads (Thermo Scientific) and washed with RIP wash buffer. Finally, the protein bound RNAs were purified using Trizol RNA extraction method and subjected to RT-qPCR.

### Co-immunoprecipitation (CO-IP) assay

Cells were collected and lysed in 0.5mL IP lysis buffer with added proteinase inhibitor. The lysate was then precleared and incubated with desired primary antibody (anti-PSPC1 and anti- β catenin antibody) at 40 C overnight. Next the antibody-protein complex was incubated with A/G dynabeads (Thermo Scientific) and incubated on rotation for 4 h at 4 °C. The bead-protein-antibody complex was then washed and eluted using Laemmli buffer followed by western blot.

### Cycloheximide (CHX) Chase assay

The cycloheximide chase assay was performed by treating the cells with translational inhibitor cycloheximide (CHX) (Sigma Aldrich) at a concentration of 50 μm for 0 h. 2 h, 4 h and 8 h. Total protein was collected from the cells at indicated time points and subjected to western blot after protein quantification. Half-life of protein was calculated in every group of cells from the densitometric analysis of the western bands.

### MG132 assay

CASC19 overexpressing and knockdown cell lines along with their control lines were treated with MG132 (20 µmol/L) (Sigma Aldrich) and incubated for 4 h. Total cellular protein from each group was collected and PSPC1 protein expression was detected by densitometric analysis of the western blot images with ImageLab software.

### Ubiquitination assay

Cells were treated with proteasome inhibitor MG132 at a concentration of 20 µmol/L for 4 h. Then the cells were collected and lysed with IP lysis buffer. The lysate was then immunoprecipitated with anti-PSPC1 antibody and anti-IgG antibody and the eluted protein was immunoblotted with anti-Ubiquitin antibody.

### Bio-informatic analysis

GEPIA2 (Gene Expression Profiling Interactive Analysis 2) database was used to get the differentially expressed lncRNAs from TCGA pancreatic cancer RNA sequencing data (Tang et al. 2019). For bio-informatic prediction of the interacting proteins of CASC19, 'beRBP’ and ‘CatRapid’ webtools were used (Agostini et al. 2013; Yu et al. 2019). Enriched pathways from the differentially expressed genes of the RNA sequencing data was done using ‘Cluster Profiler’ package of R (Yu et al. 2012).

### Statistical analysis

All the experiments were repeated at least three times. Values measured were presented as mean ± standard deviation and comparisons of the values between two groups were performed using Student’s paired t-test. *p*-value < 0.05 was considered to indicate a statistically significant difference between the groups. GraphPad Prism was used for data illustration.

##  Results

### Elevated CASC19 expression was found to be associated with pancreatic cancer progression and reduced patient survival rates

**Fig. 1:**
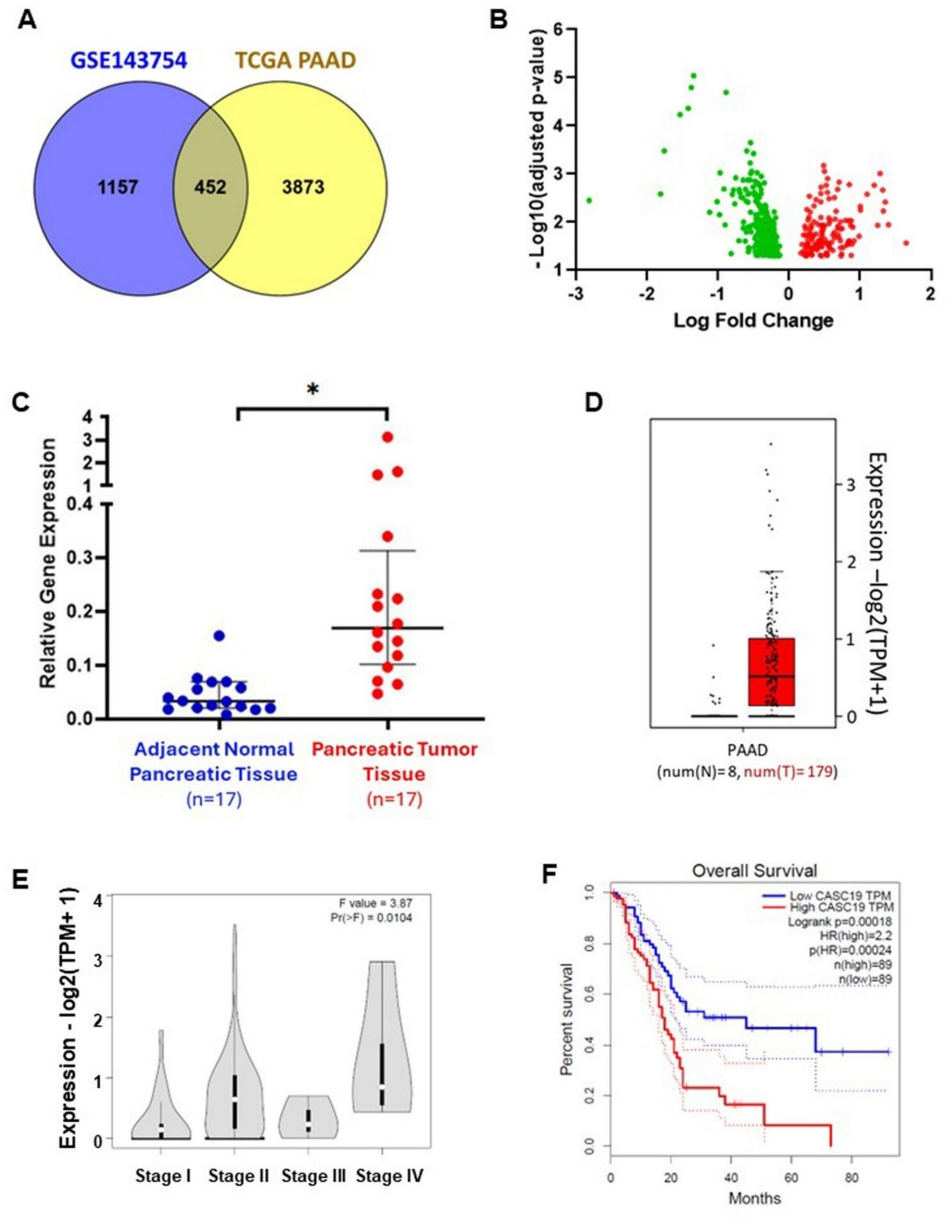
CASC19 overexpression is correlated with advanced cancer and poor prognosis in pancreatic cancer. **A** Venn diagram showing the overlapping differentially expressed lncRNAs of microarray data and TCGA RNA seq data of pancreatic cancer patients. **B** Volcano plot for the common DE-lncRNAs in pancreatic cancer. Upregulated genes are marked in red and downregulated genes are marked in green colour. **C** Expression quantification of CASC19 from qRT-PCR shows significant upregulation in pancreatic cancer tissue (*n*=17) compared to adjacent normal pancreatic tissue (*n*-17). ‘*’indicates *p*-value < 0.05. **D** Relative expression of CASC19 in pancreatic tumor tissue and normal pancreatic tissue from GEPIA2 TCGA-PAAD RNA-seq dataset. **E** Violin plot showing association of CASC19 expression with different stages of pancreatic cancer. **F** Kaplan–Meier plot showing pancreatic cancer patients with high CASC19 expression predicts poor overall survival and disease-free survival that the patients with low CASC19 expression

From our previous study, we have taken the list of differentially expressed lncRNAs (DElncRNAs) in pancreatic cancer. The study included microarray data of 11 pancreatic tumor tissue and 9 adjacent normal pancreatic tissue (GSE143754) which resulted in 1609 significant differentially expressed lncRNAs (adjusted *p*-value 0.05) with 364 upregulated and 1245 downregulated lncRNAs (Chhatriya et al. 2020). Alongside, we have also taken the DElncRNAs of TCGA pancreatic cancer RNA sequencing data from GEPIA2 (Gene Expression Profiling Interactive Analysis 2) database. Differential analysis from TCGA RNA sequencing data yielded 4321 DElncRNAs (adjusted *p*-value 0.05; 3281 upregulated and 1040 downregulated lncRNAs). Next, the two sets of DElncRNAs from two different platforms were compared and only the common DElncRNAs were considered as the lncRNAs altered in pancreatic cancer (452 DElncRNAs, 140 upregulated and 312 downregulated lncRNA) (Fig. 1A) (Supplementary Table-S4). Volcano plot for these common DElncRNAs have been shown in Fig. 1B. Subsequently, we wanted to focus on the oncogenic lncRNAs of pancreatic cancer and therefore it was decided to proceed with the top 25 upregulated lncRNAs (according to the fold change values from the microarray data) for further exploration. CASC19 (Cancer susceptibility candidate 19) was one such lncRNA which caught our attention as it was found to be deregulated in several other cancer types (Liu et al. 2020, 2021; Qu et al. 2019; Wang et al. 2019a, b, 2020, 2023a, b, c; Zhao et al. 2019) and encoded by chromosomal locus 8q24.21, a “gene desert” and mutational hotspot region, linked to numerous cancer phenotypes (Wilson and Kanhere 2021). Regarding the status of CASC19 in pancreatic cancer, there was only a single report delineating its role in pancreatic cancer through microRNA sponging (Lu et al.^ 2020^), with no other detailed mechanisms known. Hence, we were curious to further look for its oncogenic role and mechanism of action in pancreatic cancer. First, the expression of CASC19 was determined in separate set of 17 pairs of pancreatic tumor tissues and adjacent normal pancreatic tissues (collected from a separate cohort of patients with tumours of either TNM Stage-IB or Stage-II) by qRT-PCR. The result showed significantly high expression of CASC19 in pancreatic cancer patients as compared to the noncancerous part of pancreas (Fig. 1D). This finding corroborated with the high CASC19 expression level in PDAC patients from RNA-sequencing data of TCGA patient cohort (Fig. 1E). We have also found elevated CASC19 level was associated with more aggressive stages of pancreatic cancer (Fig. 1F), according to the web server GEPIA2. The Kaplan–Meier overall survival (OS) analysis from GEPIA2 database depicted that pancreatic cancer patients with high CASC19 expression exhibited significantly poorer survival than the PDAC patients with low expression of CASC19 (Fig. 1G). These data assumed a strong contribution to CASC19 in the development and progression of pancreatic cancer.

### Effect of CASC19 on pancreatic cancer cell proliferation, cell cycle and apoptosis

**Fig. 2:**
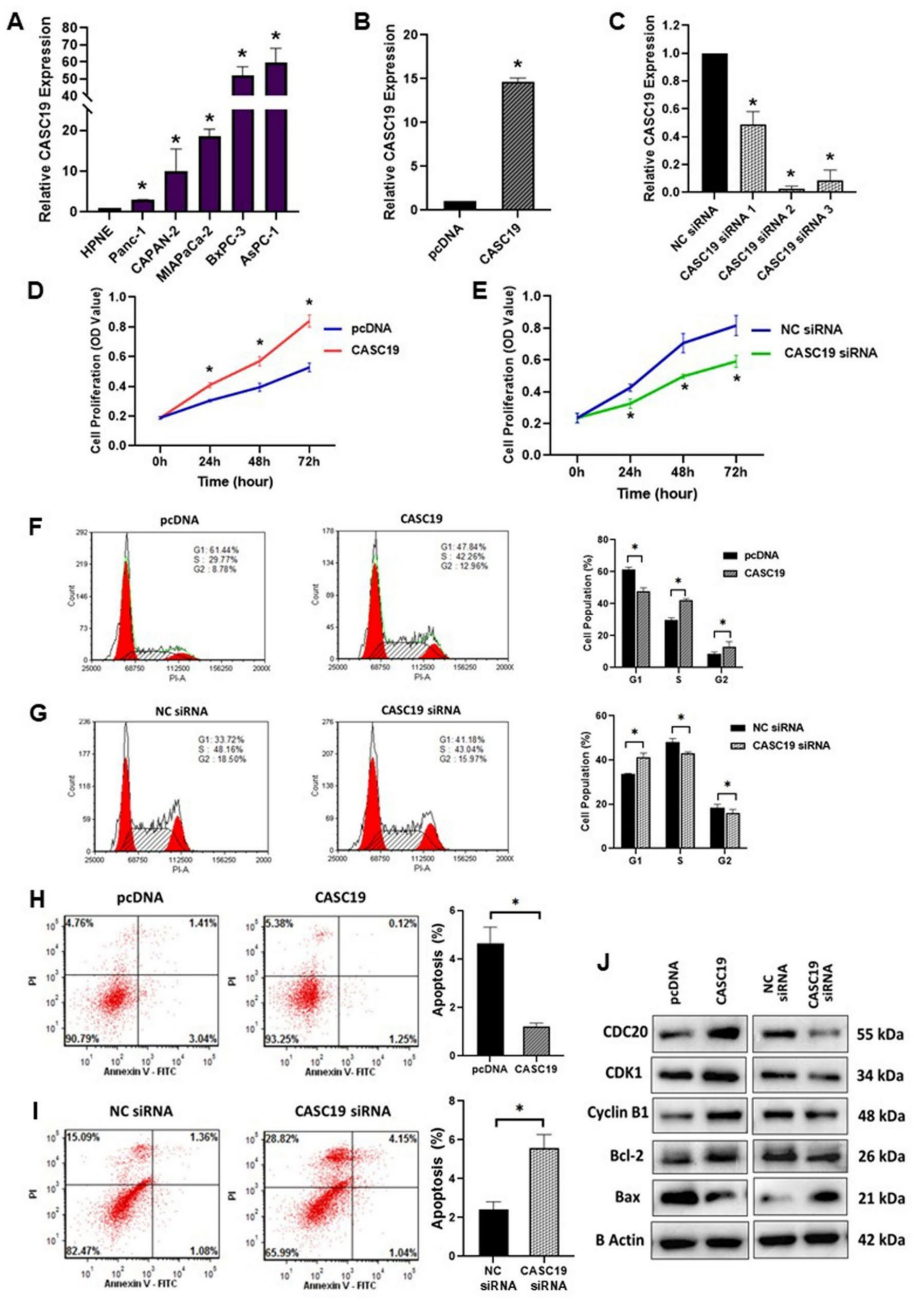
CASC19 promotes proliferation and cell cycle progression and suppress apoptosis of pancreatic cancer cell. **A** Relative CASC19 expression levels in five pancreatic cancer cell lines (Panc-1, CAPAN-2, MIAPaCa-2, BxPc-3 and AsPc-1) and one normal pancreatic ductal epithelial cell line (control cells) detected by RT-qPCR. **B** Quantification of CASC19 expression by qRT-PCR in stably CASC19 overexpressed MIAPaCa-2 cells.** C** qRT-PCR results showed the efficiency of CASC19 knockdown in MIAPaCa-2 cells transfected with siRNAs**. D** Cell viability of CASC19 overexpressed cells detected by MTT assay. OD value was measured at 570nm. **E** Cell viability of CASC19 knockdown cells detected by MTT assay. OD value was measured at 570nm.** F** Effect of CASC19 overexpression on cell cycle as assessed by PI staining and flow cytometry.** G** Effect of CASC19 knockdown on cell cycle as assessed by PI staining and flow cytometry.** H** Investigating the effect on cellular apoptotic phenotype upon CASC19 overexpression and **I**) knockdown. **J** Western blot analysis depicts the protein expression of genes involved in cell cycle and apoptosis after CASC19 overexpression and knockdown**. **For all the panels, ‘*’indicates *p*-value< 0.05

Gene expression level of CASC19 was determined in several human pancreatic cancer cell lines (AsPc-1, BxPc-3, MIAPaCa-2, Panc-1 and CAPAN-2) and one normal human pancreatic ductal cell line (hTERT-HPNE) using qRT-PCR. CASC19 expression was found to be much higher in the pancreatic cancer cell lines than the normal pancreatic ductal cell line (Fig. 2A). Amongst these five pancreatic cancer cell lines, MIAPaCa-2 had the intermediate expression level of CASC19 and thus we chose the cell line for further investigations and cellular/functional assays. Therefore, we overexpressed and silenced CASC19 expression in the MIAPaCa-2 cell line by stable and transient transfection respectively and the CASC19 expression levels were verified using qRT-PCR in the transfected cells (Fig. 2B, C). This was followed by some cellular assays to determine the oncogenic effect of CASC19 on pancreatic cancer progression.

The proliferation of pancreatic cancer cells was notably enhanced by CASC19 overexpression (Fig. 2D) and reduced by downregulation of CASC19, as measured by MTT assay (Fig. 2E). Cell cycle assay showed that CASC19 overexpression exhibited a prolonged S phase with a shorter G1 phase, thus promoting cell cycle whereas CASC19 knockdown aided cell cycle arrest (Fig. 2F and G). Flow cytometry-based apoptosis assay demonstrated that percentage of apoptotic cells was markedly diminished in CASC19 overexpressing cell lines compared to their vector control groups (Fig. 2H) and there was a significant elevation in number of cells undergoing apoptosis in CASC19 silenced MIAPaCa-2 cells (Fig. 2I). Consistent with these observations, it was also found that overexpression of CASC19 increased the protein expression of cell cycle markers CDC20, CDK1 and cyclin B1, whereas expression of these proteins decreased with silencing of CASC19, as evidenced by western blotting. Furthermore, CASC19 overexpression led to an increase in expression of anti-apoptotic protein Bcl-2 and a reduction in the expression of pro-apoptotic protein Bax. Similarly, protein expression of Bcl-2 decreased and Bax increased when CASC19 was silenced in pancreatic cancer cell line MIAPaCa-2 (Fig. 2J). These findings inferred that high CASC19 expression intensifies pancreatic cancer progression through modulating cell proliferation, cell cycle and apoptosis.

### CASC19 promotes pancreatic cancer cell migration

**Fig. 3:**
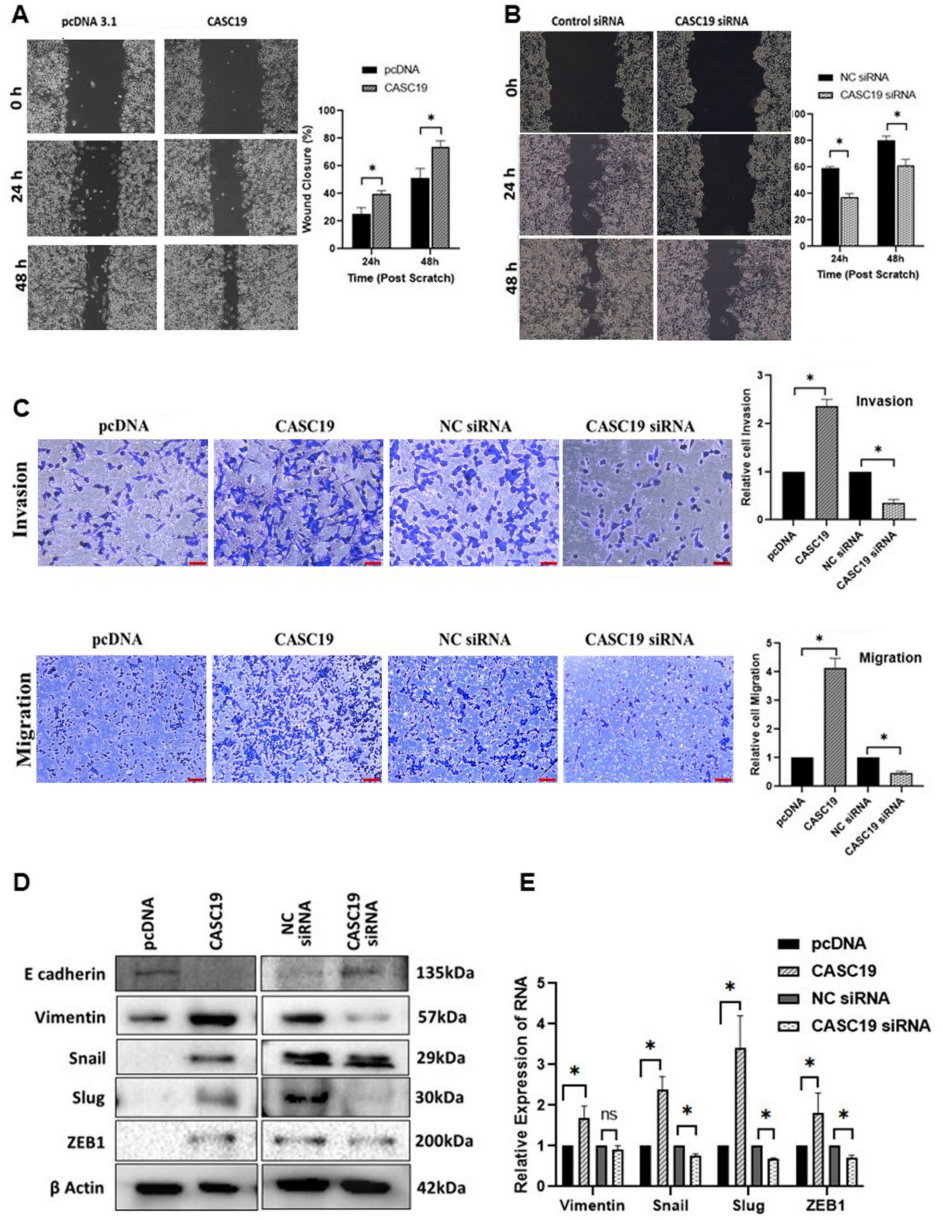
Alteration in CASC19 expression governs the migratory trait of cell in pancreatic cancer **A** Wound healing assay determines that overexpression of CASC19 boosts migratory ability of pancreatic cancer cell, whereas. **B** depletion of CASC19 inhibits the same. ‘*’indicates *p*-value< 0.05. **C** CASC19 overexpression and knockdown affect the migration and invasion property of cancer cells as determined by the transwell assay.‘*’indicates *p*-value < 0.05. **D** Western blot analysis depicts the protein expression of genes involved in EMT after CASC19 overexpression and knockdown. **E** qRT-PCR results showing the RNA expression pattern of EMT marker genes upon CASC19 expression alteration**. **‘*’indicates *p*-value < 0.05

Metastatic potential of a cancer cell is generally determined by the migratory and invasive properties of the cell, and we wanted to investigate whether changes in CASC19 expression could modulate the phenomenon. The wound-healing assay revealed that CASC19 overexpressing cells showed a significant increase in wound-closure rate than the control cells (Fig. 3A) and a decreased rate of wound-healing was seen in cells with the CASC19 knockdown, suggesting restricted motility (Fig. 3B). Transwell assay further confirmed that CACS19 overexpression led to an augmented migratory and invasive potential in pancreatic cancer cell line MIAPaCa-2. However, a reduced migration and invasion was seen in pancreatic cancer cell line with loss of expression of the CASC19 (Fig. 3C). Collectively, these findings suggest that elevated expression of CASC19 promote metastasis in pancreatic cancer cells. Considering the key role of the epithelial–mesenchymal transition (EMT) in metastasis, we investigated whether CASC19 could influence the process of EMT by analyzing the protein and mRNA levels of various EMT-related markers. As illustrated in Fig. 3D, overexpression of CASC19 led to an increase in the mesenchymal marker Vimentin, Snail, Slug, Claudin and ZEB1, while the expression of epithelial marker E-cadherin decreased. In support of this observation, expression of mesenchymal markers was decreased, and epithelial markers were found to be increased with CASC19 knockdown (Fig. 3D). RNA expression of the mesenchymal markers was also changed according to their protein expression upon CASC19 overexpression and knockdown (Fig. 3E). Overall, these findings demonstrate that elevated expression of CASC19 contributes to EMT and metastasis in pancreatic cancer cells.

### CASC19 being a nuclear LncRNA modulates the global transcriptome in pancreatic cancer

**Fig. 4:**
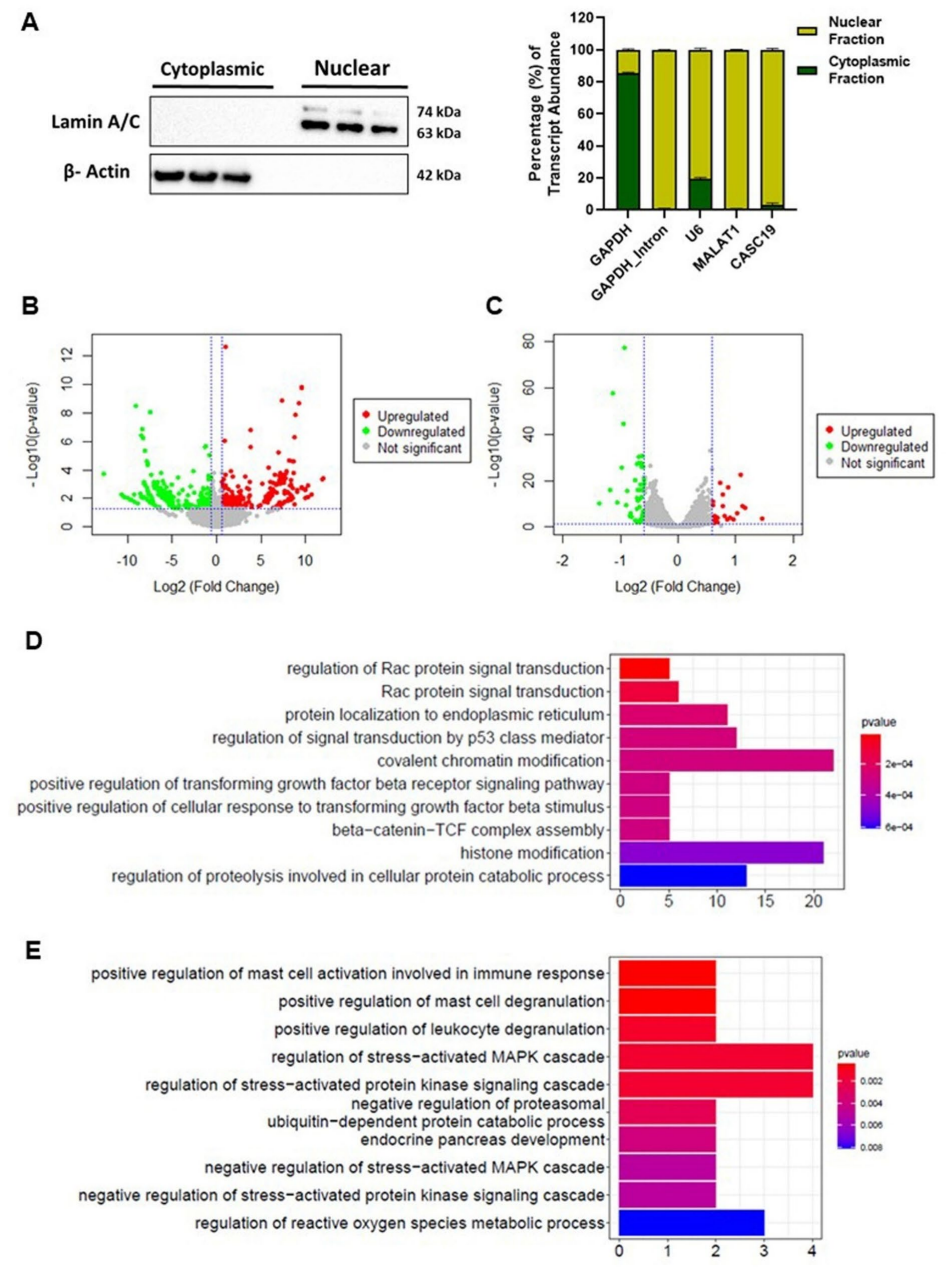
CASC19, a nuclear lncRNA, exerts a modulatory effect on the global transcriptome in pancreatic cancer.** A** Nuclear and cytoplasmic abundance of CASC19 expression (left and right panel).** B** Volcano plot representing the differentially expressed genes when CASC19 is overexpressed. **C** Volcano plot showing the differentially expressed genes upon CASC19 knockdown.** D** Pathway enrichment analysis of DEGs in CASC19 overexpressed pancreatic cancer cell. **E** Pathways enriched in CASC19 depleted pancreatic cancer cell indicates alteration in MAPK-cascade and ubiquitin dependent protein catabolic process

To gain insights into the molecular mechanism of the lncRNAs, determining their subcellular localization is very crucial (Carlevaro-Fita and Johnson 2019). Therefore, nuclear and cytoplasmic fractionation followed by qRT-PCR revealed the presence of CASC19 in the nuclear fraction of the pancreatic cancer cell MIAPaCa-2 (Fig. 4A). Nuclear localization of the lncRNA CASC19 indicated that it might contribute towards progression of pancreatic cancer via transcriptional regulation of its target genes. Thus, to find out the CASC19 regulated transcriptome, CASC19 was overexpressed and knocked down in pancreatic cancer cell line MIAPaCa-2 and subjected to RNA-sequencing. RNA-sequencing analysis identified 464 differentially expressed genes (228 upregulated and 236 downregulated) upon CASC19 overexpression (Fig. 4B) (Supplementary Table-S5) and 76 DEGs (28 upregulated and 48 downregulated) (*p*-value < 0.05, Fold Change +/−1.5) on CASC19 knockdown (Fig. 4C) (Supplementary Table-S6). Pathway analysis identified that positive regulation of TGF-β signaling pathway and β-Catenin-TCF complex assembly are the two important oncogenic pathways mediated by CASC19 overexpression along with other pathways like Rac protein signal transduction, protein localization to endoplasmic reticulum, signal transduction by p53 class mediator, chromatin and histone modification etc. (Fig. 4D). Oncogenic pathways affected by CASC19 downregulation are MAPK cascade, proteasomal ubiquitin-dependent protein catabolic process etc. (Fig. 4E). Taken together, these results suggested that modulation of CASC19 expression regulates the transcription of several oncogenic pathway related genes.

### CASC19 interacts with a nuclear protein PSPC1 and increases its stability

**Fig. 5:**
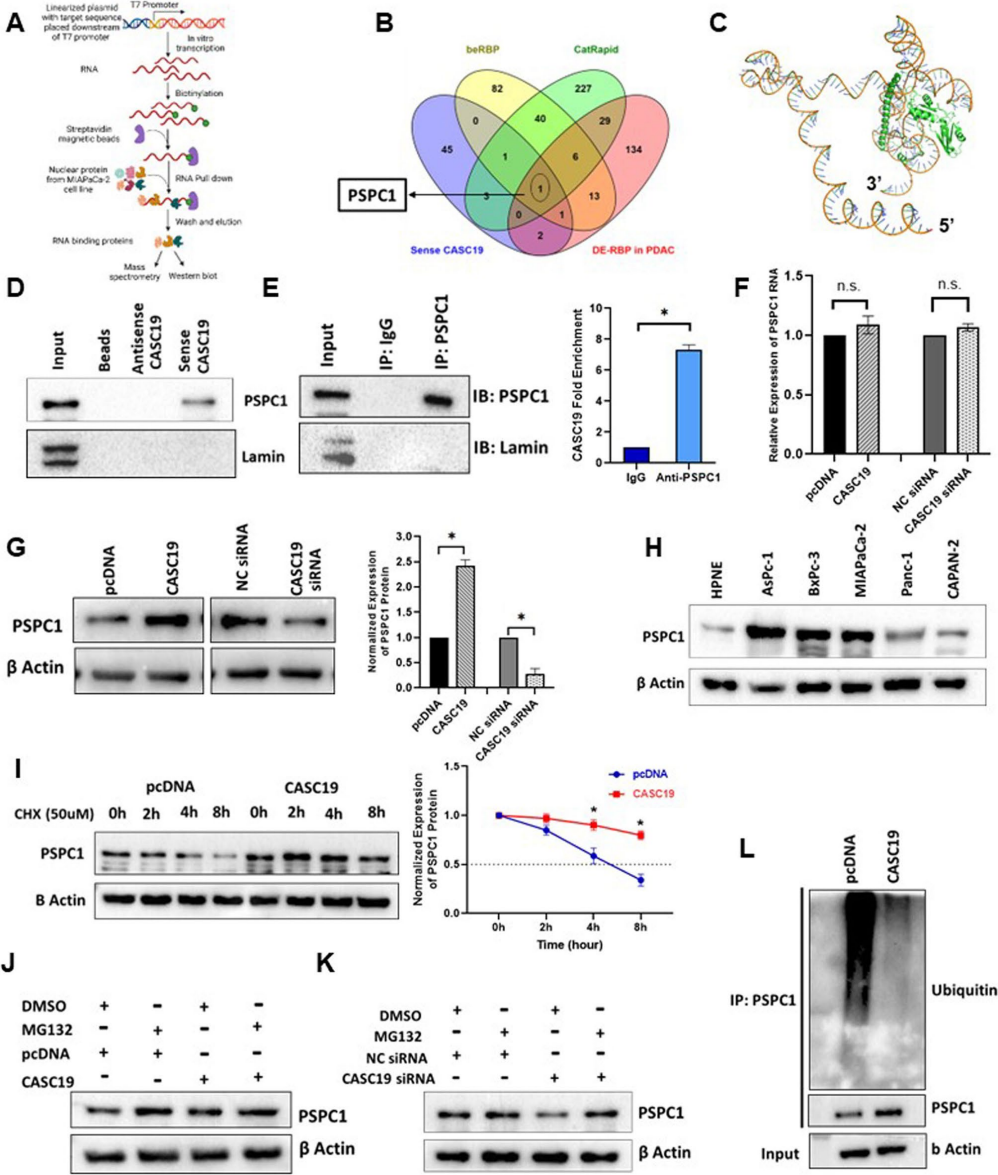
CASC19 binds to PSPC1 and increases its stability in pancreatic cancer cells.** A** Conceptual diagram of the biotin-pull down assay. **B** Venn diagram representing the RNA binding proteins common to the CASC19-pull down mass-spectrometry data, RBP prediction from CatRapid, beRBP and RNA binding proteins altered in PDAC derived from a meta-analysis. **C** Docking simulation of the RBP-lncRNA interaction model using HADDOCK software. Monomeric PSPC1 protein structure is shown in green. **D** Streptavidin RNA pull down assay of full-length sense and antisense CASC19 transcript and PSPC1 immunoblotting. **E** RNA immunoprecipitation followed by qRT-PCR showing CASC19 RNA binding with PSPC1 protein. ‘*’indicates *p*-value < 0.05. **F** Q-PCR result showing the RNA expression of PSPC1 when CASC19 is overexpressed or downregulated. **G** Western blot image of PSPC1 portraying the PSPC1 expression upon CASC19 expression alteration. ‘*’indicates *p*-value < 0.05. **H** PSPC1 expression analysis by western blot in pancreatic cancer cell lines compared to normal pancreatic cell lines. **I** Western blot analysis reveals PSPC1 protein level when cells were treated with cycloheximide (CHX, 50uM) for the indicated time, upon CASC19 overexpression. ‘*’indicates *p*-value< 0.05 **J** Western blotting showing PSPC1 expression in CASC19 overexpressed and (K) depleted cells after treatment with MG132 and DMSO for 4 hours. **L** Western blot analysis of PSPC1 ubiquitination in MG132 treated MIAPaCa-2 cells with CASC19 overexpression

Long noncoding RNAs exert their functions primarily by interacting with RNA binding proteins to regulate various cellular processes. Thus, to elucidate the mechanism of action of CASC19 and identify its interacting proteins, we performed an RNA pull down assay followed by mass-spectrometry. Sense and antisense transcripts of CASC19 were transcribed in-vitro, biotinylated and incubated with nuclear lysate of pancreatic cancer cell MIAPaCa-2 and the pulled protein extracts were then subjected to mass-spectrometry (Fig. 5A). A total of 53 proteins were found to be bound specifically to the sense CASC19 transcript (Supplementary Table-S7). In parallel, we used two bioinformatic tools, CatRapid’ and ‘beRBP’ to predict the interacting proteins for CASC19 and from there we predicted 307 (number of RNA Binding Domains Instances ≥ 3) (Supplementary Table-S8) and 144 CASC19 interacting proteins (Supplementary Table-S9) respectively. Additionally, we wanted to consider only The RBPs deregulated in pancreatic cancer and thus we have taken 186 deregulated RBPs in pancreatic cancer from our previous study (Mukherjee and Goswami 2021). Combining all this information we got only one common protein PSPC1 (Paraspeckle Component-1) as a potential CASC19 binding protein (Fig. 5B). Next, we wanted to bioinformatically validate the lncRNA-RBP interaction through RNA-protein molecular docking and thus with the HADDOCK’ and ‘HDOCK’ software we have determined their interaction with their possible molecular structures (Supplementary Figure-S1). The first ranked structure form HADDOCK (HADDOCK score = −41.3) was selected after critical inspection as it has maximum number of residues interacting with lncRNA and is placed between two structural segments of the RNA (Fig. 5C). Interaction of CASC19 and PSPC1 was then experimentally validated through Western-blotting of the eluted protein from RNA pull down where PSPC1 was detected only in input and sense CASC19 sample, but not in antisense and beads control (Fig. 5D). The interaction was further confirmed by RNA immunoprecipitation (RIP) using anti-PSPC1 antibody (Fig. 5E). Evidence from the above experiments proves that PSPC1 directly interacts with the lncRNA CASC19. To get a sense of the interaction between CASC19 and PSPC1, expression of PSPC1 was examined at both RNA and protein level after CASC19 overexpression and knockdown. PSPC1 RNA expression was not affected by the CASC19 expression alteration (Fig. 5F). However, the protein level of PSPC1 was found to be increased with high CASC19 expression and vice versa (Fig. 5G). PSPC1 protein expression was also high in pancreatic cancer cell lines like that of CASC19 expression (Fig. 5H). Thus, it can be perceived that CASC19 might modulate PSPC1 expression through translational regulation or post translational regulation such as affecting its protein stability. Accordingly, cycloheximide (CHX) chase assay was performed to detect whether CASC19 can influence PSPC1 protein expression at translational level. Thus, cells undergone CHX treatment, inhibiting de novo protein synthesis and it was found that half-life of PSPC1 was increased when CASC19 was upregulated, indicating a positive impact on PSPC1 stability, a crucial mode of post translational regulation (Fig. 5I). Ubiquitin proteasome mediated degradation is one of the mainstream pathways for protein degradation (Zhan et al. 2023) and here we wanted to verify whether interaction with CASC19 have an impact on this pathway for influencing PSPC1 protein stability. Thus, pancreatic cancer cells with upregulated and downregulated CASC19 were treated with a proteasome inhibitor MG132 and it was found that PSPC1 protein expression remained stable in CASC19 depleted and control cancer cells and the expression level was higher in CASC19 overexpressing and vector control cells (Fig. 5J, Fig. 5K). Furthermore, ubiquitination assay was carried out in CASC19 overexpressing and control MIAPaCa-2 cells treated with MG132 and amount of ubiquitinated PSPC1 was found to be less in CASC19 upregulated cells (Fig. 5L). As we have previously seen that, expressions of both CASC19 and PSPC1 were high in pancreatic cancer cell line MIAPaCa-2 than normal pancreatic cell line HPNE, we compared the amount ubiquitinated PSPC1 in both the cell lines and have found that ubiquitination of PSPC1 was remarkably less in MIAPaCa-2 cells which has high endogenous CASC19 expression. Thus, to further specify that PSPC1 ubiquitination is regulated by CASC19 expression, we overexpressed CASC19 in HPNE cell line where basal CASC19 expression is less than any pancreatic cancer cells and it was found that the amount of ubiquitinated PSPC1 was less in CASC19 overexpressed HPNE cells (Supplementary Figure-S2). Therefore, it can be concluded that CASC19 expression did not exert any influence on PSPC1 protein synthesis, rather post-translationally regulate PSPC1 expression by affecting its ubiquitin-mediated degradation and enhance PSPC1 protein stability in pancreatic cancer.

### Knockdown of PSPC1 partially recovered the oncogenic phenotypes influenced by CASC19 overexpression

**Fig. 6:**
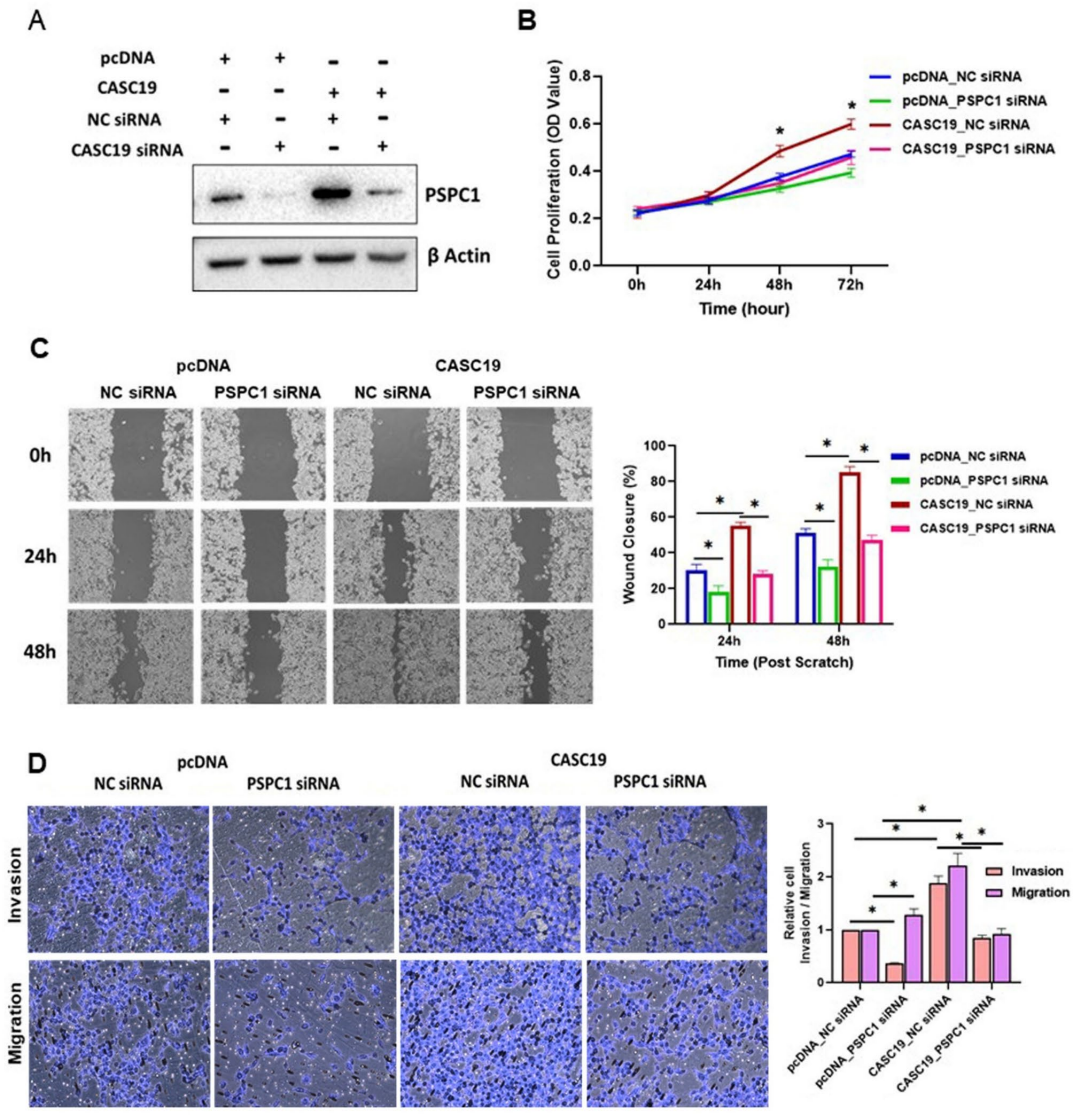
Oncogenic phenotypes induced by CASC19 overexpression rescued partly by PSPC1 knockdown. **A** Western blotting showing PSPC1 expression in MIAPaCa-2 cells treated with denoted transfection. **B**-**D** MTT, wound-healing, transwell assay for cell proliferation in MIAPaCa-2 cells co-transfected with CASC19 plasmid and PSPC1 siRNA. OD value was measured at 570nm. ‘*’indicates *p*-value< 0.05

To further confirm that CASC19-induced oncogenic functions are mediating by PSPC1 in pancreatic cancer, we performed a rescue experiment in CASC19 overexpressing cells with siRNA against PSPC1. Western-blot assay of the rescue experiment showed that upregulated PSPC1 protein level induced by CASC19 overexpression was abolished by PSPC1 siRNA in pancreatic cancer cell MIAPaCa-2 (Fig. 6A). Outcome of MTT assay demonstrated that PSPC1 silencing could reverse the increased proliferation in pancreatic cancer cells caused by CASC19 overexpression (Fig. 6B). Moreover, elevated wound-healing, migration and invasion ability of cancer cells with CASC19 upregulation were significantly attenuated by PSPC1 siRNA in respect to control cells (Fig. 6C, D). Hence, it can be concluded that CASC19 induced tumorigenic traits are driven by PSPC1 protein.

### CASC19 drives pancreatic cancer progression by maintaining nuclear β-Catenin abundance through PSPC1

**Fig. 7:**
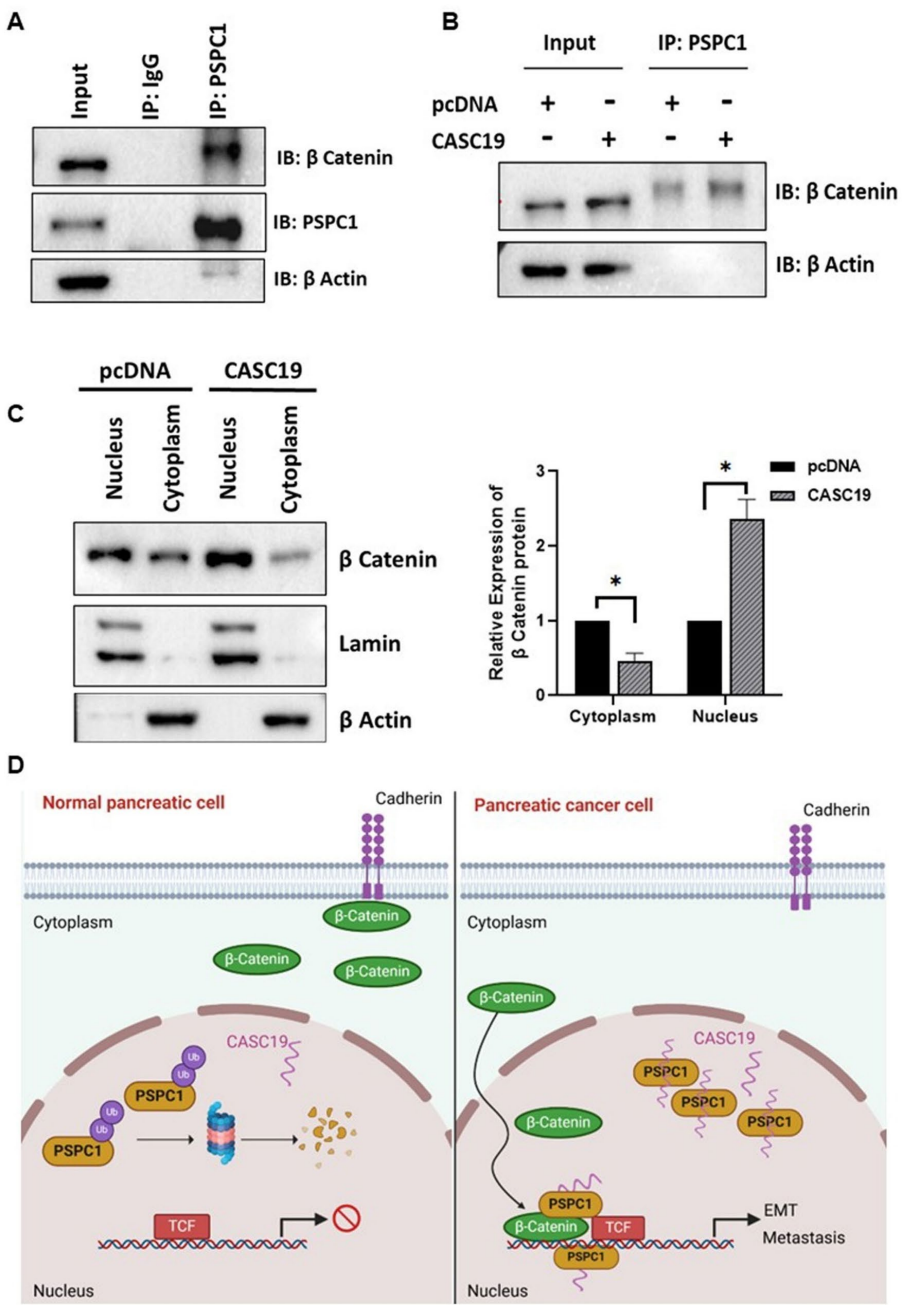
CASC19-PSPC1-β Catenin axis promotes oncogenicity through nuclear retention of β Catenin.** A** Coimmunoprecipitation with anti-PSPC1 antibody and immunoblotting for β-catenin showing PSPC1-β Catenin interaction. **B** Coimmunoprecipitation of PSPC1 and immunoblotting with β-catenin in CASC19 overexpressed condition. **C** Nuclear and cytoplasmic distribution of β-Catenin upon CASC19 overexpression.‘*’indicates *p*-value < 0.05. **D** Schematic representation of the CASC19 mediated PSPC1 stability and cancer progression: Elevated expression of CASC19 in pancreatic cancer cells resulted in increased PSPC1 protein stability, subsequently leading to higher nuclear retention of β-Catenin and promotion of pancreatic cancer metastasis

Literature suggests that PSPC1 interacts with β-Catenin and maintains the nuclear retention of β-Catenin to promote the transcription of EMT and metastasis-related genes via wnt-β-Catenin pathway in hepatocellular carcinoma (Lang et al. 2019). Hence, we wanted to investigate whether a similar mechanism is contributing to pancreatic cancer progression, as our findings, so far, showed that high CASC19 expression contributes to more PSPC1 protein stability and more PSPC1 availability in the nucleus. Accordingly, we showed the interaction between PSPC1 and β-Catenin by co-immunoprecipitation (Fig. 7A, Supplementary Figure-S3). CASC19 overexpressing cells have also shown higher β-Catenin expression upon co-immunoprecipitation with anti-PSPC1 antibody (Fig. 7B), which indicated more PSPC1/β-Catenin interaction in CASC19 overexpressing condition. Next, we examined the subcellular localization of β-Catenin upon CASC19 overexpression, and the results showed that the amount of nuclear β-Catenin was higher in CASC19 overexpressing cell line than the vector control with a reduced β-Catenin level in the cytoplasmic fraction (Fig. 7C). Therefore, these findings summarize that increased availability of PSPC1 protein due to high CASC19 expression resulted in increased nuclear retention of β-Catenin, a potent transcription factor for several oncogenes to vitalize pancreatic cancer progression (Fig. 7D).

##  Discussion

Worldwide, pancreatic cancer is recognized as one of the most prevalent and lethal forms of gastrointestinal malignancies with a poor understanding of the mechanism underlying tumorigenesis and disease progression (Li et al. 2022). Evidence from numerous studies have shown that lncRNAs are the critical regulators of several tumorigenic processes/hallmarks (Bhan et al.^ 2017^; Li et al. 2017; Zhou et al. 2022). A range of lncRNAs also has been shown to play critical roles in driving pancreatic tumor growth, advancement and progression/metastasis (Cheng et al. 2015; Li et al. 2016; Wang et al. 2023a, b, c; Yoshimura et al. 2018). However, oncogenic property and mechanistic investigation of a majority of deregulated lncRNAs in pancreatic cancer are still unknown. In our current study, we have identified a lncRNA CASC19 which is notably upregulated in pancreatic tumor tissue and pancreatic cancer cell lines and is associated with advanced stages of the disease and poor patient survival. We have also found that high CASC19 expression promotes pancreatic cancer cell proliferation, cell cycle progression and metastatic ability of the cell with a reduction in apoptosis. These findings featured potent oncogenicity of CASC19 during pancreatic cancer development. Thus, further understanding of the role of CASC19 in pancreatic malignancy can gain insight into its molecular mechanisms and identify promising therapeutic approaches.

CASC19 has been the focus of many recent studies, revealing its aberrant expression and functional roles across multiple cancers (Liu et al. 2020, 2021; Qu et al. 2019; Wang et al. 2019a, b, 2020, 2023a, b, c; Zhao et al. 2019). In line with earlier discoveries, our study corroborated that CASC19 functions as a pro-oncogenic factor. Nevertheless, the precise mechanisms underlying CASC19’s impact on pancreatic cancer progression remains to be elucidated.

There is a broad consensus that subcellular localization of lncRNAs is the primary determinant of their molecular activities (Carlevaro-Fita and Johnson 2019; Bridges et al. 2021). Gene regulation through microRNA sponging was the major mode of action for CASC19 in cancer development, as evidenced by the prior reports (Wang et al. 2019a, b, 2020; Zhang et al. 2024). To delve deeper into the mechanisms of CASC19 in pancreatic cancer progression, we carried out a subcellular fractionation assay and found that CASC19 is present mainly in the nucleus of pancreatic cancer cells. Regulation of gene expression is a cardinal function of nuclear lncRNAs through chromatin organization, DNA damage response, transcriptional regulation, RNA processing, etc. (Song et al. 2021). In this study, we have identified the differentially expressed genes upon CASC19 overexpression and knockdown through RNA sequencing and also identified the affected pathways. Positive regulation of TGF-β signaling pathway is one of the significant pathways upon CASC19 overexpression which is a crucial mediator of EMT and metastasis (Colak and Ten Dijke 2017; Gough et al. 2021; Syed 2016). Beta-catenin TCF complex assembly is another pathway activated upon CASC19 upregulation which is associated with Wnt/β-catenin signaling, a versatile pathway involved in transcription of many oncogenes (Shang et al. 2017; Song et al. 2024). Chromatin modification is another important pathway associated with CASC19 overexpression which is also responsible for several tumorigenic processes (Nair and Kumar 2012; Zhao et al. 2021). Regulation of proteasomal ubiquitin-dependent protein catabolic process is one of the crucial pathways affected upon CASC19 silencing which might alter the stability of numerous tumor-suppressive and oncogenic proteins (Liu et al. 2024).

Subcellular distribution of lncRNAs largely determine their molecular interaction network for potential functional mechanism (Zhang et al. 2014). Expanding research indicates interaction of nuclear lncRNAs with RBPs contributes critically in driving cancer progression (Huang et al. 2021; Yao et al. 2022). Therefore, we investigated the interacting proteins of CASC19 by RNA pulldown assay followed by mass-spectrometry and other bioinformatics approaches. We have identified the binding of CASC19 with a pro-metastatic nuclear protein PSPC1 and again validated the interaction by RIP assay. LncRNAs may influence lncRNA-protein interactions by modulating protein stability, altering their intracellular distribution, or serving as structural frameworks. Recent findings suggest that CASC19 supports gastric cancer progression by hindering proteasomal degradation of its binding protein CREB1, thus increasing its stability (Wang et al. 2023a, b, c). Among several aspects of lncRNA-protein interaction, we have found that CASC19 affects PSPC1 protein stability without affecting its expression at transcript level. Therefore, we conducted CHX assay, MG132 assay and ubiquitination assay which suggests that CASC19 increases PSPC1 protein stability by reducing its ubiquitin-proteasome mediated degradation in pancreatic cancer. Similar findings were also shown by another report where lncRNA LOC105369504 alters PSPC1 protein expression in colorectal cancer (Zhan et al. 2023).

Paraspeckle component protein PSPC1 is found to be upregulated in breast cancer, lung cancer, nasopharyngeal cancer and hepatocellular carcinoma along with pancreatic cancer (Zhan et al. 2023; He et al. 2021; Takeiwa et al.^ 2022^; Yuan et al. 2024). Recent finding identifies the role of PSPC1 as a contextual determinant of subcellular translocation of oncogenes associated with growth, EMT and metastasis of cancer cells (Mohankumar et al.^ 2022^). Moreover, PSPC1 upregulation facilitates EMT, stemness, and metastasis by driving cytoplasmic translocation of active PTK6 and nuclear sequestration of oncogenic β-catenin. The resulting PSPC1/β-catenin complex thus augments oncogenic transcription via Wnt3a autocrine signaling (Lang et al. 2019). Similarly, in our present study, we have established that high CASC19 expression increases PSPC1 protein availability in the nucleus which promotes nuclear retention of β-catenin leading to tumor progression. This observation also corroborates with the finding that CASC19 upregulation potentiates the metastatic pathway via TGF-β signaling and β-catenin TCF complex assembly. Via successive rescue experiments, it was authenticated that the oncogenic functions of CASC19 in pancreatic cancer are mediated by regulating PSPC1 expression.

Despite the insights gained, there are certain limitations in our study. Firstly, we have not determined the specific site where CASC19 binds to PSPC1. Secondly, while our findings demonstrated the involvement of the ubiquitin-proteasome system in the regulation of PSPC1 by CASC19, the ligase involved in the ubiquitination of PSPC1 has not been identified. Also, the precise mechanism through which CASC19 restrains the ubiquitination of PSPC1 remains unexplored. Therefore, further research is needed to uncover the detailed molecular mechanisms of pancreatic carcinogenesis mediated by CASC19-PSPC1 axis.

## Conclusion

To summarize, our results imply that CASC19 is an oncogenic lncRNA that promotes tumor proliferation, cell cycle, EMT and metastasis in pancreatic cancer with a reduction in apoptosis and is linked to poor prognosis in pancreatic cancer. CASC19 achieves its oncogenic effects by facilitating PSPC1 protein stability via reduction of its proteasome-dependent degradation and favouring nuclear sequestration of β-catenin. Accompanied with continued research, the novel CASC19/PSPC1/β-catenin axis holds promise as a potential and effective therapeutic target for advanced pancreatic cancer.

## Supplementary Information

Supplementary Material 1: Supplementary Figure-S1. Molecular docking structures of CASC19-PSPC1 interaction. A Predicted model of lncRNA CASC19. A total of 129 homologous sequences were used to cover the entire length of the lncRNA which consists of several stem and loop regions with a large part of the RNA forming stable tertiary structure. B Crystal structure of PSPC1 (PDB Id: 5IFN). Two monomers of the dimeric structure are shown in green and gray ribbons. The amino acids present at the dimeric interface are colored yellow and presented in sticks. C The structure of the CASC19-PSPC1 complexes along with dock score (HDOCK score) for docking using HDOCK. D The four best docked structure of CASC19-PSPC1 interaction from HADDOCK with their respective dock scores. E The 2D interaction diagram of the interaction between PSPC1 and CASC19. The hydrogen bonds between amino acids and nucleic acids are shown by green dotted lines. The residues and nucleotides marked by semi circles are involved in non-polar interaction

Supplementary Material 2: Supplementary Figure-S2. CASC19 overexpression decreases PSPC1 ubiquitination in normal pancreatic HPNE cell line. **A **Western blot analysis of PSPC1 ubiquitination in MG132 treated HPNE and MIAPaCa-2 cells. B Western blot analysis of PSPC1 ubiquitination in MG132 treated HPNE cells with CASC19 overexpression

Supplementary Material 3: Supplementary Figure-S3. Co-immunoprecipitation β-catenin showing PSPC1-β Catenin interaction. Co-immunoprecipitation with anti-β-catenin antibody and immunoblotting for PSPC1 showing PSPC1-β Catenin interaction in pancreatic cancer cell line MIAPaCa-2

Supplementary Material 4: Supplementary Table-S1 A comprehensive list of primers employed in this study for q-PCR

Supplementary Material 5: Supplementary Table-S2 Information of the siRNAs used in this study for knockdown

Supplementary Material 6: Supplementary Table-S3 The details of the antibodies used in this study

Supplementary Material 7: Supplementary Table-S4 Common DEGs in pancreatic cancer from a microarray data and TCGA-RNA sequencing data

Supplementary Material 8: Supplementary Table-S5 Differentially expressed genes upon CASC19 overexpression in pancreatic cancer cell line MIAPaCa-2

Supplementary Material 9: Supplementary Table-S6 Differentially expressed genes upon CASC19 knockdown with siRNA in pancreatic cancer cell line MIAPaCa-2

Supplementary Material 10: Supplementary Table-S7 List of 53 RNA binding proteins obtained from RNA-pull down and mass-spectrometry that bound exclusively to sense strand of CASC19

Supplementary Material 11: Supplementary Table-S8 RNA binding proteins predicted to be bound to CASC19 as per CatRapid database

Supplementary Material 12: Supplementary Table-S9 RNA binding proteins predicted to be bound to CASC19 from beRBP database

## Data Availability

The data has been submitted to the Indian Biological Data Centre with the IBDC Study accession number INRP000259.

## Abbreviations

LncRNA: Long non-coding ribonucleic acid
ZEB1: Zinc Finger E-Box Binding Homeobox 1
TGF-β: Transforming Growth Factor Beta
CREB1: CAMP Responsive Element Binding Protein 1
