# Supplementary figures and images for: LncRNA CASC19 promotes pancreatic cancer progression by increasing PSPC1 protein stability and facilitating the oncogenic PSPC1/ β-Catenin pathway
Source: Mol Med. 2025 Sep 29;31:305. doi: 10.1186/s10020-025-01363-7 (PMC12482102; doi:10.1186/s10020-025-01363-7)

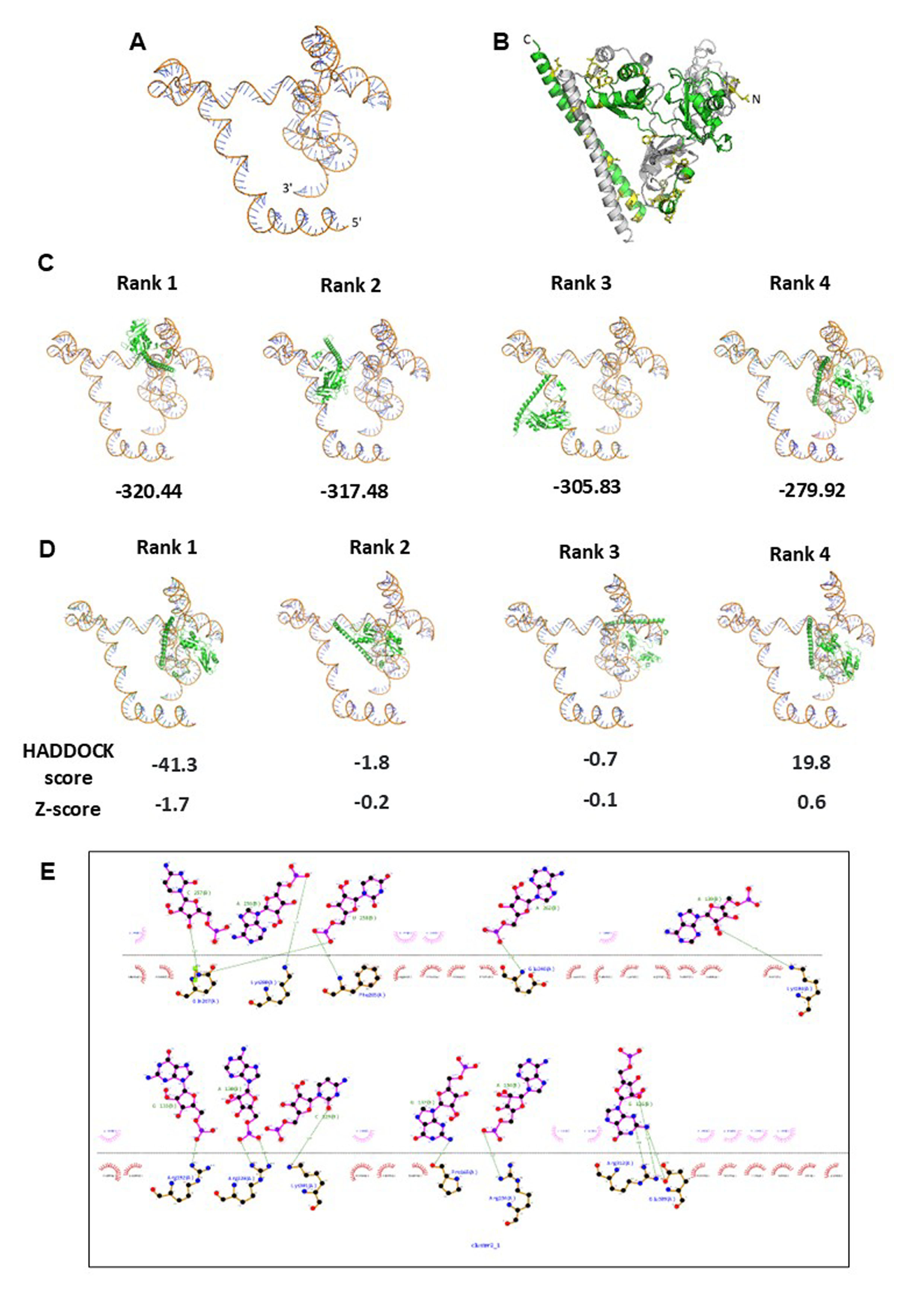

Supplement: Supplementary file 1 — Supplementary Material 1: Supplementary Figure-S1. Molecular docking structures of CASC19-PSPC1 interaction. A Predicted model of lncRNA CASC19. A total of 129 homologous sequences were used to cover the entire length of the lncRNA which consists of several stem and loop regions with a large part of the RNA forming stable tertiary structure. B Crystal structure of PSPC1 (PDB Id: 5IFN). Two monomers of the dimeric structure are shown in green and gray ribbons. The amino acids present at the dimeric interface are colored yellow and presented in sticks. C The structure of the CASC19-PSPC1 complexes along with dock score (HDOCK score) for docking using HDOCK. D The four best docked structure of CASC19-PSPC1 interaction from HADDOCK with their respective dock scores. E The 2D interaction diagram of the interaction between PSPC1 and CASC19. The hydrogen bonds between amino acids and nucleic acids are shown by green dotted lines. The residues and nucleotides marked by semi circles are involved in non-polar interaction [file 10020_2025_1363_MOESM1_ESM.jpg]

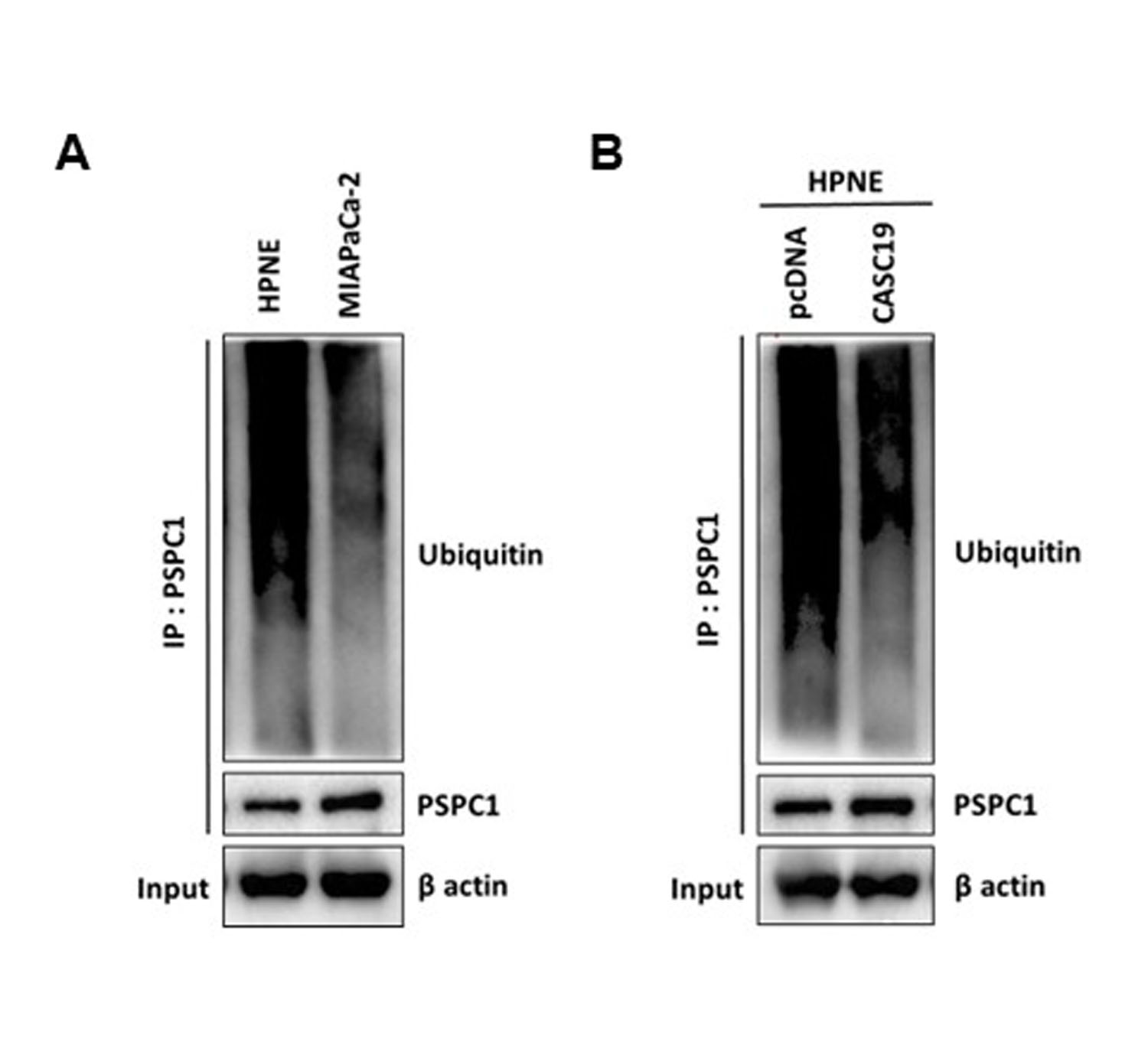

Supplement: Supplementary file 11 — Supplementary Material 11: Supplementary Table-S8 RNA binding proteins predicted to be bound to CASC19 as per CatRapid database [file 10020_2025_1363_MOESM11_ESM.jpg]

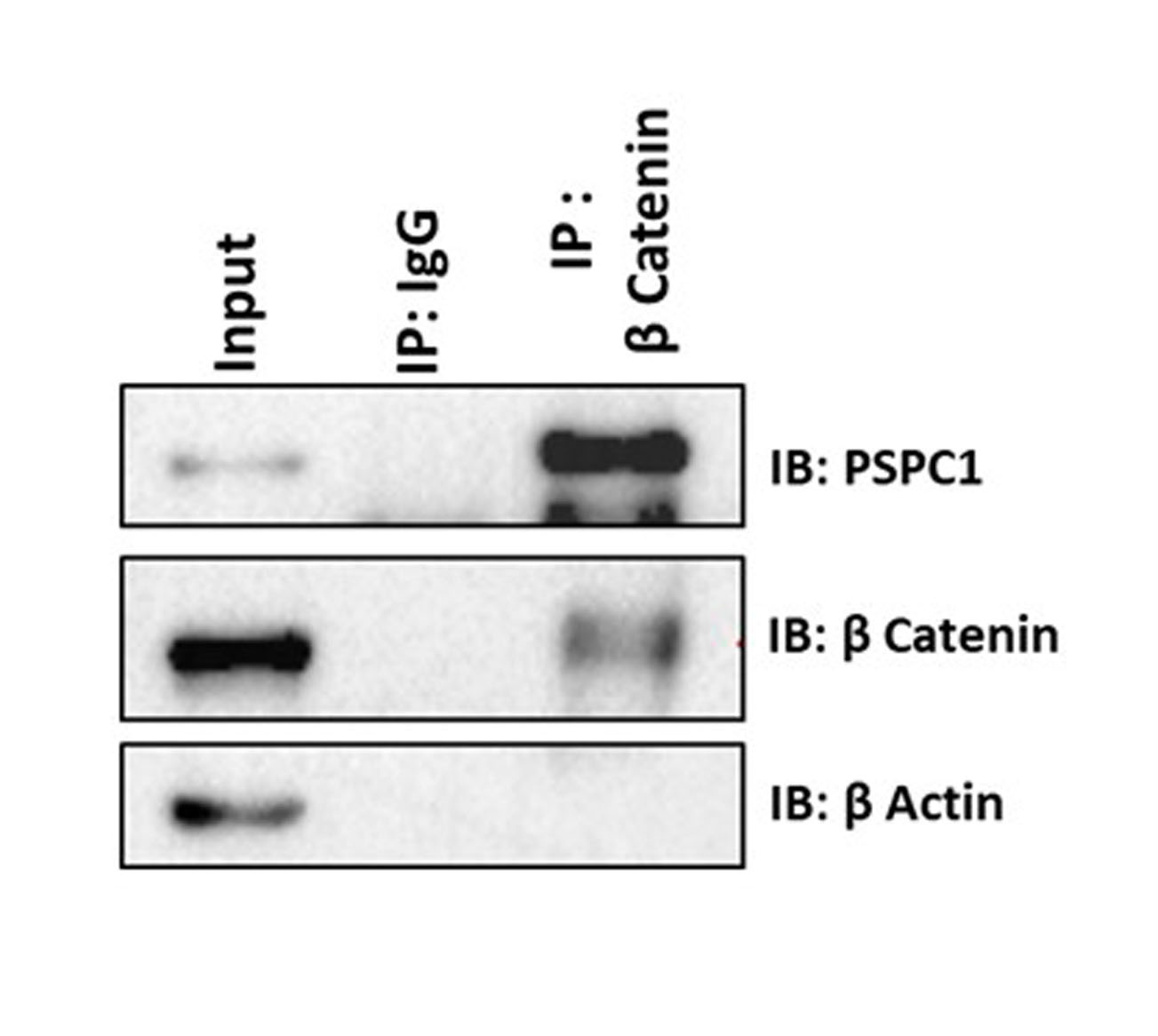

Supplement: Supplementary file 12 — Supplementary Material 12: Supplementary Table-S9 RNA binding proteins predicted to be bound to CASC19 from beRBP database [file 10020_2025_1363_MOESM12_ESM.jpg]
