# Supplementary material for: LncRNA CASC19 promotes pancreatic cancer progression by increasing PSPC1 protein stability and facilitating the oncogenic PSPC1/ β-Catenin pathway
Source: Mol Med. 2025 Sep 29;31:305. doi: 10.1186/s10020-025-01363-7 (PMC12482102; doi:10.1186/s10020-025-01363-7)
Supplement: Supplementary file 2 — Supplementary Material 2: Supplementary Figure-S2. CASC19 overexpression decreases PSPC1 ubiquitination in normal pancreatic HPNE cell line. A Western blot analysis of PSPC1 ubiquitination in MG132 treated HPNE and MIAPaCa-2 cells. B Western blot analysis of PSPC1 ubiquitination in MG132 treated HPNE cells with CASC19 overexpression [file 10020_2025_1363_MOESM2_ESM.docx]

**Supplementary Table 1: siRNA sequences**

| **Name** | **Sequence Positions** | **Target sequence**  **(5’-3’)** |
| --- | --- | --- |
| CASC19_siRNA1 | 160-185 | CTAACAAAGTTGACCTTAGAATTGG |
| CASC19_siRNA2 | 222-247 | ACTGCATGCTTCTGATGTGAGTTCA |
| CASC19_siRNA3 | 17-42 | CATACTACATTGAAATTATTTCCTTA |
| PSPC1_siRNA1 | 614-634 | AAGCTGTTGTGGTTGTGGATG |
| PSPC1_siRNA2 | 357-377 | CAAGAGGCTCTTCGAACGCTA |
