# Supplementary material for: LncRNA CASC19 promotes pancreatic cancer progression by increasing PSPC1 protein stability and facilitating the oncogenic PSPC1/ β-Catenin pathway
Source: Mol Med. 2025 Sep 29;31:305. doi: 10.1186/s10020-025-01363-7 (PMC12482102; doi:10.1186/s10020-025-01363-7)
Supplement: Supplementary file 3 — Supplementary Material 3: Supplementary Figure-S3. Co-immunoprecipitation β-catenin showing PSPC1-β Catenin interaction. Co-immunoprecipitation with anti-β-catenin antibody and immunoblotting for PSPC1 showing PSPC1-β Catenin interaction in pancreatic cancer cell line MIAPaCa-2 [file 10020_2025_1363_MOESM3_ESM.docx]

**Supplementary Table 2: Primer sequences**

| **Name** | **Forward primer (5’-3’)** | **Reverse primer (5’-3’)** |
| --- | --- | --- |
| CASC19 | GAGGAAGGCAGCACAATGATG | CTTGCCAGTGTCTTCTCCTGA |
| PMM1 | CAGAAGCTACGAAGTAGAGTGCAGA | CCGTTCTCGGCAAACACATA |
| PSPC1 | CTTGCCAGAGAAGCTGATGCAG | CAAGAGCCTTCCATCGAGATGC |
| GAPDH | GGGAGCCAAAAGGGTCAT | GAGTCCTTCCACGATACCAA |
| GAPDH Intron | GTGCTACATGGTGAGCCCCAAA | GGGGAATACGTGAGGGTATGAAG |
| U6 | CTCGCTTCGGCAGCACATATACT | ACGCTTCACGAATTTGCGTGTC |
| MALAT1 | AGCCCAAATCTCAAGCGGTGC | TGCATCGAGGTGAGGGGTGA |
| Vimentin | TGTCCAAATCGATGTGGATGTTTC | TTGTACCATTCTTCTGCCTCCTG |
| Snail | TTTACCTTCCAGCAGCCCTA | CCCACTGTCCTCATCTGACA |
| Slug | GGGGAGAAGCCTTTTTCTTG | TCCTCATGTTTGTGCAGGAG |
| ZEB1 | TTACACCTTTGCATACAGAACCC | TTTACGATTACACCCAGACTGC |
