# Supplementary material for: LncRNA CASC19 promotes pancreatic cancer progression by increasing PSPC1 protein stability and facilitating the oncogenic PSPC1/ β-Catenin pathway
Source: Mol Med. 2025 Sep 29;31:305. doi: 10.1186/s10020-025-01363-7 (PMC12482102; doi:10.1186/s10020-025-01363-7)
Supplement: Supplementary file 4 — Supplementary Material 4: Supplementary Table-S1 A comprehensive list of primers employed in this study for q-PCR [file 10020_2025_1363_MOESM4_ESM.docx]

**Supplementary Table 3: Antibody Information**

| **Antibody** | **Company** | **Catalog Number** | **Specificity** | **Dilution**  **in western** |
| --- | --- | --- | --- | --- |
| E Cadherin | Cell Signalling technology | 3195S | Rabbit | 1:500 |
| Vimentin | Abclonal | A19607 | Rabbit | 1:2000 |
| Snail | Cell Signalling technology | 38795S | Rabbit | 1:500 |
| Slug | Cell Signalling technology | 9585S | Rabbit | 1:500 |
| Claudin | Cell Signalling technology | 13255S | Rabbit | 1:500 |
| ZEB1 | Cell Signalling technology | 3396S | Rabbit | 1:1000 |
| PSPC1 | Abcam | Ab104238 | Rabbit | 1:1000 |
| Lamin | Cell Signaling Technology |  | Mouse | 1:2000 |
| β-catenin | BD bioscience | 610153 | Mouse | 1:1000 |
| β-actin | Santa Cruze | SC47778 | Mouse | 1:1000 |
| Ubiquitin | Cell Signaling Technology | 58395S | Rabbit | 1:1000 |
| IgG | Cell Signaling Technology | 5415S | Mouse | NA |
| IgG | Abclonal | AC005 | Rabbit | NA |
| Anti-rabbit IgG, HRP-linked Antibody | Cell Signaling Technology | 7074S | Goat | 1:3000 |
| HRP-conjugated Goat anti-Mouse IgG (H+L) | Abclonal | AS003 | Goat | 1:10000 |
